# Internet Addiction, Symptoms of Anxiety, Depressive Symptoms, Stress Among Higher Education Students During the COVID-19 Pandemic

**DOI:** 10.3389/fpubh.2022.893845

**Published:** 2022-06-14

**Authors:** Beata Gavurova, Samer Khouri, Viera Ivankova, Martin Rigelsky, Tawfik Mudarri

**Affiliations:** ^1^Department of Addictology, First Faculty of Medicine, Charles University and General University Hospital in Prague, Prague, Czechia; ^2^Institute of Earth Resources, Faculty of Mining, Ecology, Process Control and Geotechnologies, Technical University of Košice, Košice, Slovakia; ^3^Department of Marketing and International Trade, Faculty of Management and Business, University of Prešov, Prešov, Slovakia

**Keywords:** mental health problems, young people, college, IAT, GAD-7, PHQ-9, PSS

## Abstract

Poor mental health is a growing concern among young people during the coronavirus disease 2019 (COVID-19) pandemic. The aim of this study was to assess the associations of Internet addiction with depressive symptoms, anxiety symptoms, and stress in higher education students during the COVID-19 pandemic, as well as to examine these mental health problems in the context of study-related characteristics. The research sample consisted of 3,099 participants from the Czech Republic (CZ: 1,422) and Slovak Republic (SK: 1,677). The Internet Addiction Test (IAT), the Generalized Anxiety Disorder (GAD-7) scale, the Patient Health Questionnaire for depressive symptoms (PHQ-9), and the Perceived Stress Scale (PSS) were used to measure mental health problems. The analyses also included demographic data (gender and age) and study-related characteristics (form of study, degree of study, field of study, distance between college and home, and housing during the semester). Based on the results of frequency and descriptive analyses, the prevalence of mental health problems was high. The most serious levels of Internet addiction (IAT cut-off point ≥ 50), to which attention should be paid, were found in 3.5% of Czech and 6.2% of Slovak students. Using the standard cut-off point of GAD-7 ≥ 10, 14.1% of Czech and 11.6% of Slovak students were identified with anxiety symptoms. Regarding the PHQ-9 with the cut-off point ≥ 10, 23.4% of Czech and 19.1% of Slovak students had depressive symptoms, which should be addressed. Using the PSS cut-off point ≥ 27, 12.9% of Czech students and 9.1% of Slovak students perceived high stress. The quantile regression analysis showed that Internet addiction was positively associated with anxiety symptoms, depressive symptoms, and stress in all of the analyzed cases (*p*-value < 0.001). In terms of study-related characteristics, the binomial logistic regression analysis revealed that risk factors for mental health problems in Czech and Slovak students were mainly full-time form of study and living away from home during the semester. Internet addiction, anxiety symptoms, depressive symptoms, and stress are issues that require increased attention, and professionals and policy-makers should implement interventions to effectively prevent and help students with psychological problems.

## Introduction

Mental health is a fundamental value that covers various areas of people's everyday lives, including psychological, emotional, and social wellbeing. It plays a crucial role in how individuals feel, think, and act. Mental health is important throughout life, and the college years are no exception. It is at this time of life that psychological problems are common and college students are often considered one of the most vulnerable population groups (1–3). This may be due to the fact that studying at college is a stressful and challenging time. Higher education students are exposed to academic pressure, many tasks, a new community, and individuation from family (4). In addition, there are many other psychological, academic, biological, lifestyle, social, and financial factors for poor mental problems among students (5–8). All of the above-mentioned facts call for increased attention to students in mental health research.

To make matters worse, the coronavirus disease 2019 (COVID-19) pandemic added to the difficulties and stressors of students (9–13). The pandemic disrupted their daily routine, exposed them to previously unknown conditions and situations, and required them to adapt to online learning. These additional challenges affected students' wellbeing and their ability to learn successfully, resulting in poor academic performance (12, 14). The seriousness of the problem is underlined by the fact that during the COVID-19 pandemic, college students reported significantly higher rates of depression, anxiety, and stress compared to the general population (15). Students' health concerns are definitely a risk factor for future psychological problems (16); following this, fear of COVID-19 has been shown in many studies to be a significant predictor of mental health disorders (17, 18). In addition to health concerns, many other factors related to lockdown have contributed to this situation, such as sudden changes in lifestyle, distant relationship with parents, lack of social support and social interaction, increased concerns on academic performance, increased concerns on financial situation, or disruptions to sleeping patterns (13, 17, 19). Changes in study context and habits also played an important role, with distance learning affecting students' mental health (14). It is also possible to note that, during the COVID-19 pandemic, internet-addicted students had lower learning satisfaction in online learning environments (20). Thus, in the context of the COVID-19 pandemic, it is also possible to speak of a mental health crisis (21). Psychological problems worsened as the lockdown time increased (22).

The COVID-19 pandemic also showed how digital technologies are intertwined with many areas of students' lives. In fact, digital technologies and the Internet provided many students around the world with important connections to university or college, fellow students, friends, and family during the long weeks to months of lockdown, as well as entertainment and distraction, which are much needed in challenging times. This underlines the positive aspect of digital technologies and the Internet, but there are also concerns about their impact on young minds (23). In fact, the time spent on these types of technologies is directly related to poor mental health (24). Even before the pandemic, evidence clearly pointed to the dark side of the Internet and its problematic use (25). In this context, Lozano-Blasco et al. (26) investigated this issue using a meta-analysis and found that Internet addiction in young adults was high in recent years. Many studies also revealed that problematic Internet usage by students was associated with mental health problems such as depression, anxiety, and stress (27–31). In other words, students with Internet addiction have a high likelihood developing mental health problems (32). In addition, it should be pointed out that Internet addiction is associated with academic burnout in college students (33). Thus, the same technologies that were useful tools in educating students during the pandemic may be a threatening element in terms of their mental health. This is the reason there is a need to strengthen the monitoring of Internet use among college students during the COVID-19 pandemic (27).

Following the above-mentioned facts, college students around the world often suffered from stress, depression, and anxiety during the COVID-19 pandemic, as evidenced by the high prevalence rates found in various studies (13, 15, 18, 34–36). These problems vary from student to student, but it is well-known that study-related characteristics such as degree of study (37–39), form of study (39, 40), field of study (3, 41–43), and distance from home and family while studying (44–47) play an important role. Evidence shows that all of the mentioned mental health problems are positively associated with inadequate-self and hated-self among college students (48). These problems can seriously damage students' mental health, and thus disrupt their educational and psychosocial functioning, as well as the future direction of their lives. The fact is that mental health problems may lead to many academic, social, and physical health consequences (49, 50). These problems are also highly related to the use of addictive substances, emphasizing the importance of the issue (51–53). In addition, students with mental health symptoms are more often isolated in their social communities, which can make their problems even worse (50). Although evidence points to low levels of stigmatizing attitudes toward psychological problems among students, to which education may have contributed, there is still a reluctance to interact with an individual who has psychological problems (54). These experiences can further exacerbate poor mental health and lead to serious consequences (55, 56).

In Czech Republic and Slovak Republic, the COVID-19 situation was critical. By comparing the pre-pandemic and pandemic periods, Hajduk et al. (57) found that there was a clear increase in mental health problems, such as depression and anxiety, among Slovak college students. Similar results have been shown in the Czech general population (58, 59). In addition to depression and anxiety (60), Slovak students also showed increased levels of stress during the pandemic (61). There is less evidence in Czech Republic, but even in that country, it was possible to speak of worrying levels of stress, depression, and anxiety during the pandemic, not only among students but also in the general population (48, 62). In fact, action is needed at the social, professional, legislative, and political levels (23, 63, 64).

The studies mentioned in the previous paragraph focused on students' mental health problems against the background of online learning during the lockdown, but studies examining the associations between the use of digital technologies, including the Internet, and psychological problems are lacking. Despite the importance of the issue, Czech Republic and Slovak Republic are neighboring countries with a common history and social priorities, where, to the knowledge of the authors of this study, no similar research can be found. Moreover, this issue is poorly addressed at a practical level in both countries (65), which is reflected in the lack of effective interventions and measures in the higher education environment. For these reasons, this study examines how Internet addiction was associated with depressive symptoms, anxiety symptoms, and perceived stress among Czech and Slovak college students during the COVID-19 pandemic, when education was predominantly distant based and online tools were used. At the same time, this study focuses on mental health problems in the context of study-related characteristics. This may provide novel and valuable findings for professionals and policy-makers in Czech Republic and Slovak Republic. Understanding the presented issue is important in the design and implementation of programs aimed at reducing and preventing mental disorders in the higher education environment during and after the pandemic.

## Materials and Methods

The aim of this study was to assess the associations of Internet addiction with depressive symptoms, anxiety symptoms, and stress in higher education students from Czech Republic and Slovak Republic during the COVID-19 pandemic, as well as to examine these mental health problems in the context of study-related characteristics. With regard to the main aim, the following research questions (RQ) and hypotheses (H) were formulated:

RQ1: Are there significant associations of Internet addiction with symptoms of depression, symptoms of anxiety, and stress among higher education students in Czech Republic and Slovak Republic?

H1a: It is assumed that there is a significant association between Internet addiction and depressive symptoms in the selected countries.

H1b: It is assumed that there is a significant association between Internet addiction and anxiety symptoms in the selected countries.

H1c: It is assumed that there is a significant association between Internet addiction and stress in the selected countries.

RQ2: Are there significant relationships of Internet addiction, symptoms of depression, symptoms of anxiety, and stress with selected study-related characteristics of Czech and Slovak students?

H2a: It is assumed that there is a significant relationship of Internet addiction, depressive symptoms, anxiety symptoms, and stress with the degree of study in the selected countries.

H2b: It is assumed that there is a significant relationship of Internet addiction, depressive symptoms, anxiety symptoms, and stress with the form of study in the selected countries.

H2c: It is assumed that there is a significant relationship of Internet addiction, depressive symptoms, anxiety symptoms, and stress with the field of study in the selected countries.

H2d: It is assumed that there is a significant relationship of Internet addiction, depressive symptoms, anxiety symptoms, and stress with housing during the semester in the selected countries.

H2e: It is assumed that there is a significant relationship of Internet addiction, depressive symptoms, anxiety symptoms, and stress with distance between home and college in the selected countries.

### Research Sample

Data collection was conducted through an electronic survey (Google Forms) during the COVID-19 pandemic in both countries. All questionnaire items were mandatory. For all forms of data collection, all information and the same instructions were provided to participants. The questionnaire was shared in Czech Republic in the Czech language and in Slovak Republic in the Slovak language. The selected scales were translated from English to Slovak. The verification of the translation was carried out by assessing the English and Slovak versions by three experts. After incorporating the comments, the scales were sent to a group of students (*n* = 20) who were asked to evaluate the comprehensibility of the items. After incorporating the students' comments, the questionnaire was translated from Slovak into Czech. The translation and clarity were assessed by three experts (assessing the English, Slovak, and Czech versions) and the comments were subsequently incorporated. The Slovak version was chosen for translation into Czech on the basis of the great similarity between the Slovak and Czech languages. The national language versions of the questionnaires are provided in Supplementary Tables 1–4.

The data collection took place in two phases. The first phase was implemented in both countries from March to May 2020, while the second phase was implemented after the summer vacation, i.e., from October to December 2020. It should be noted that ~80% of all data were collected in both countries during the first phase. In the first phase, college and university authorities (deans, vice deans, study officers) as well as student councils were contacted by emails and asked to provide a questionnaire to students with a request to complete it. In this phase, the questionnaire was also shared on social media not only in an organic way (free sharing in student groups and on fan pages) but also in a paid way (paid advertising). In the second phase, emails were sent to teachers (addresses were obtained from publicly available contact databases) asking them to share the questionnaire with students of specific study fields at individual colleges and universities. The purpose was to complete the planned sample structure. In the data collection process, the effort was to obtain a research sample as representative as possible with respect to the population under study. Sample characteristics were fitted on the basis of two main criteria. Ensuring an appropriate representation of colleges and universities was a primary concern, and thus 80% of all colleges and universities in both countries were included in the research. At the same time, fields of study were controlled in order to obtain a reasonable proportion of study fields with a minimum of 30 observations.

In the data cleaning process, the following numbers of statistical units were excluded from the sample: 179 (5.3%)—based on a control item in the questionnaire to filter out “automated” responses (agreeing that one million has six zeros, while a numerical value of 1,000,000 was also given); 27 (0.8%)—based on the identification of a system error in the recording of responses; 87 (2.6%)—based on students' foreign nationality (the research was focused on domestic students only). The final research sample consisted of 3,099 participants (Czech Republic = 1,422; Slovak Republic = 1,677). Some identifying characteristics were incorrectly reported by the participants (e.g., 1,000 as the year of birth); therefore, these responses were deleted and treated as missing data in the analyses. Table 1 presents the sample structure separately for Czech Republic (CZ) and Slovak Republic (SK).

**Table 1:** Sample structure.

**Variable**	**CZ**			**SK**		
	***n***	**%**	**% Without missing**	***n***	**%**	**% without missing**
**Gender**
Male	349	24.5	24.5	606	36.1	36.1
Female	1,073	75.5	75.5	1,071	63.9	63.9
**Age**
≤20	193	13.6	13.6	206	12.3	12.3
21–25	891	62.7	62.7	1,239	73.9	74.1
26–30	171	12.0	12.0	143	8.5	8.5
≥31	166	11.7	11.7	85	5.1	5.1
Missing	1	0.1	–	4	0.2	–
**Degree of study**
1st degree	658	46.3	46.3	1,140	68.0	68.0
2nd degree	380	26.7	26.7	428	25.5	25.5
Combined 1st and 2nd degree	50	3.5	3.5	41	2.4	2.4
3rd degree	334	23.5	23.5	68	4.1	4.1
**Form of study**
Full-time	1,041	73.2	73.2	1,550	92.4	92.4
Part-time	381	26.8	26.8	127	7.6	7.6
**Field of study**
Education	277	19.5	19.5	80	4.8	4.8
Humanities & Arts	101	7.1	7.1	78	4.7	4.7
Social, Economic & Legal Sciences	665	46.8	46.8	671	40.0	40.0
Natural Science	50	3.5	3.5	73	4.4	4.4
Design, Technology, Production & Communications	93	6.5	6.5	164	9.8	9.8
Agricultural & Veterinary Sciences	67	4.7	4.7	53	3.2	3.2
Health Service	54	3.8	3.8	180	10.7	10.7
Services (tourism, sports, security, transport, logistics)	69	4.9	4.9	240	14.3	14.3
Informatics, Mathematics & ICT	46	3.2	3.2	138	8.2	8.2
**Housing during the semester**
Dormitory	243	17.1	17.1	702	41.9	41.9
Rented accommodation	287	20.2	20.2	139	8.3	8.3
Living with relatives	202	14.2	14.2	68	4.1	4.1
Living with a friend	40	2.8	2.8	30	1.8	1.8
At home	650	45.7	45.7	738	44.0	44.0
**Distance between home and college**
≤20.0 kilometers	461	32.4	32.4	400	23.9	24.0
20.1–50.0 kilometers	318	22.4	22.4	357	21.3	21.4
50.1–100.0 kilometers	349	24.5	24.5	424	25.3	25.5
≥100.1 kilometers	294	20.7	20.7	485	28.9	29.1
Missing	0	–	–	11	0.7	–

*CZ, Czech Republic; SK, Slovak Republic; n, frequency; ICT, Information and Communication Technologies*.

### Ethical Considerations

All aspects of the research were conducted in adherence to the Declaration of Helsinki. The study was approved by the General University Hospital in Prague as individual research (Ref. 915/20 S–IV). Informed consent to participate in the research was provided by all study participants, who received adequate information about the study objectives, required data, and expected benefits. The participants were informed that their responses would not be judged and their confidentiality would be respected. They were also informed that all participation was confidential, anonymous, voluntary, and harmless. The participants did not receive any financial reward. Also, all study participants completed the questionnaire consisting of demographic data, items assessing Internet addiction, items assessing anxiety symptoms, items assessing depressive symptoms, and items assessing perceived stress.

### Measures

The analyses included four measures of mental health (anxiety symptoms, depressive symptoms, stress, Internet addiction) based on existing scales presented in previous studies. The study conducted by Kroenke et al. (66) provided measures of anxiety and depressive symptoms. These were the *Generalized Anxiety Disorder* (GAD-7) as a 7-item scale, and the *Patient Health Questionnaire* for depressive symptoms (PHQ-9) as a 9-item scale. Both scales offered the following possible responses: (0) not at all, (1) several days, (2) more than half the days, and (3) nearly every day. The total GAD-7 score was the sum of the coded responses and its ranges indicated: no anxiety symptoms (0–4), mild anxiety symptoms (5–9), moderate anxiety symptoms (10–14), and severe anxiety symptoms (15 and more). The severity ranges of the total PHQ-9 score were as follows: no depressive symptoms (0–4), mild depressive symptoms (5–9), moderate depressive symptoms (10–14), moderately severe depressive symptoms (15–19), and severe depressive symptoms (20 and more).

Another measure was the *Perceived Stress Scale* (PSS) developed by Cohen et al. (67). This scale consisted of 10 items and offered the following responses: (0) never, (1) almost never, (2) sometimes, (3) fairly often, and (4) very often. The total PSS score was the sum of the coded responses and its ranges indicated low stress (0–13), moderate stress (14–26), and high stress (27–40).

The last fourth measure was the *Internet Addiction Test* (IAT) based on an existing scale developed by Young (68). This 20-item scale offered possible responses using a Likert scale: (0) not applicable to your life, (1) rarely, (2) occasionally, (3) frequently, (4) often, and (5) always. The total IAT score could take on a value in ranges indicating no addiction (a normal level of Internet usage) (0–30), mild addiction (31–49), moderate addiction (50–79), and severe addiction (80 and more). Based on the above-mentioned, the higher the total score, the more severe the mental health problem.

The level of reliability (Cronbach's alpha) was acceptable in all of the analyzed cases (i.e., higher than 0.7). For GAD-7, it was 0.878 in the Czech sample and 0.865 in the Slovak sample. For PHQ-9, it was 0.867 in the Czech sample and 0.861 in the Slovak sample. For PSS, it was 0.757 in the Czech sample and 0.708 in the Slovak sample. For IAT, it was 0.888 in the Czech sample and 0.899 in the Slovak sample.

### Statistical Procedure

The statistical analysis of the data consisted of several analytical procedures. First, frequency and descriptive analyses were used to provide a first look at the data using statistical measures such as mean, standard deviation, 25th percentile, median, 75th percentile, minimum, and maximum. Non-parametric tests of differences were also applied when assessing the descriptive dimension of the data. The Mann-Whitney *U*-test was used for differences between two categories and the Kruskal-Wallis *H*-test was used for differences between three or more categories. In subsequent analytical steps, the quantile regression analysis (with the 25th, 50th, and 75th percentiles) was used to evaluate the associations between Internet addiction (IAT) and mental health problems such as anxiety symptoms (GAD-7), depressive symptoms (PHQ-9), and stress (PSS). In addition, the binomial logistic regression analysis was chosen to assess the relationships between selected study-related characteristics and Internet addiction, anxiety symptoms, depressive symptoms, and stress in higher education students. In this case, the IAT, GAD-7, PHQ-9, and PSS scores were adjusted to a binomial form, with 0 representing the lowest range and 1 representing the other higher ranges of the total scores.

## Results

This section presents the results of the analytical processing, organized according to the applied analysis. The section begins with a statistical description of the data. Subsequently, it presents the results of a quantile regression analysis evaluating the associations between Internet addiction and anxiety symptoms, depressive symptoms, and stress. This analysis was used to answer RQ1. It concludes by examining the relationships between selected study-related characteristics and Internet addiction, depressive symptoms, anxiety symptoms, and stress among higher education students during the COVID-19 pandemic. The logistic regression analysis was used to answer RQ2.

All analyses were sorted by country as Czech Republic and Slovak Republic are different entities (Czech Republic is a more developed country), despite their common history. Also, the Mann-Whitney U test revealed significant differences between these countries in all variables except IAT (*p*-value: PHQ-9 = 0.0015, GAD-7 = 0.0005, PSS = 0.0061, IAT = 0.2562).

Table 2 shows the results of the descriptive analysis and difference tests of the total scores for Internet addiction (IAT), anxiety symptoms (GAD-7), depressive symptoms (PHQ-9), and stress (PSS). With a focus on IAT, it can be pointed out that a score below 30 means no Internet addiction. Based on this, it was possible to conclude that Czech and Slovak students did not report addiction problems, as their mean scores were lower than 30 (IAT mean: CZ = 25.13 ± 12.46; SK = 26.01 ± 13.45). In both countries, the upper quartile (75th percentile) indicated that 25% of students reported mild or more severe problem behaviors related to Internet addiction. The maximum values indicated that the problem of Internet addiction was very serious for some students. In terms of GAD-7, a score higher than four indicates mild anxiety symptoms, while the mean scores for Czech and Slovak students were slightly above this threshold (GAD-7 mean: CZ = 4.71 ± 4.61; SK = 4.15 ± 4.26). However, a median score was 3 in both countries, indicating that no anxiety symptoms were present. Based on the 75th percentile, it was possible to state that 25% of students reported mild or more severe anxiety symptoms. The maximum values of the total GAD-7 score achieved in both countries also indicated severe anxiety symptoms in some students. When focusing on PHQ-9, it was possible to confirm that Czech and Slovak students reported mild depressive symptoms, as their mean total scores were in a range from 5 to 9 (PHQ-9 mean: CZ = 6.34 ± 5.50; SK = 5.77 ± 5.29). Higher scores were also found for stress, where a range of 14–26 indicated moderate stress. The mean PSS score was 19.82 ± 5.57 in Czech Republic and 19.28 ± 5.27 in Slovak Republic.

**Table 2:** Results of descriptive analysis and difference tests.

	**CZ**				**SK**			
	**IAT**	**GAD-7**	**PHQ-9**	**PSS**	**IAT**	**GAD-7**	**PHQ-9**	**PSS**
Mean	25.13	4.71	6.34	19.82	26.01	4.15	5.77	19.28
Standard deviation	12.46	4.61	5.50	5.57	13.45	4.26	5.29	5.27
25th percentile	16.0	1.0	2.0	16.0	16.0	1.0	2.0	16.0
Median	24.0	3.0	5.0	20.0	24.0	3.0	4.0	19.0
75th percentile	33.0	7.0	9.0	24.0	34.0	6.0	8.0	22.0
Minimum	0	0	0	4	0	0	0	2
Maximum	81	21	27	38	90	21	27	39
**Test of differences and median measure across sample categories**							
**Gender** (Diff.)	179,496^*^	140,009.5	157,274^*^	127,457.5^*^	280,752^*^	280,983^*^	309,466.5	256,159^*^
Male	25	2	4	17	26.5	2	4	18
Female	24	4	5	20	23	3	4	20
**Age** (Diff.)	67.87^*^	25.28^*^	49.09^*^	52.69^*^	22.92^*^	15.78^*^	31.66^*^	18.68^*^
≤20	25	4	7	21	26	3	5	20
21–25	25	4	5	20	24	3	4	19
26–30	21	3	4	19	25	3	4	19
≥31.00	17	2	3	17	18	1	2	17
**Degree of study** (Diff.)	6.99	4.28	8.61^*^	11.53^*^	2.18	2.92	2.45	4.80
1st degree	24	3	5	20	24	3	4	19
2nd degree	23	3	4	19	24	3	4	19
Combined 1st and 2nd degree	27.5	4	7	21	27	2	3	18
3rd degree	24.5	4	5	20	23	3.5	5	19
**Form of study** (Diff.)	153,938.5^*^	178,619.5^*^	166,294.5^*^	165,358^*^	78,613.5^*^	87,018.5^*^	78,542^*^	84,908^*^
Full-time	25	4	5	20	25	3	4	19
Part-time	20	3	4	19	19	2	3	17
**Field of study** (Diff.)	22.97^*^	32.50^*^	39.63^*^	47.89^*^	16.68^*^	13.99	18.38^*^	11.89
Education	24	4	6	21	23	3	4	19
Humanities & Arts	24	4	4	19	26.5	4	5.5	20
Social, Economic & Legal Sciences	23	3	4	19	24	3	4	19
Natural Science	27	3	4	19.5	24	2	4	19
Design, Technology, Production & Communications	22	2	3	16	24	2.5	4.5	18
Agricultural & Veterinary Sciences	28	5	9	22	25	4	6	19
Health Service	23.5	5	6	20	23.5	3	4.5	19
Services (tourism, sports, security, transport, logistics)	23	4	4	19	25	3	4	19
Informatics, Mathematics & ICT	30.5	2	5	19.5	28	3	5	19
**Housing during the semester** (Diff.)	39.74^*^	6.86^*^	19.87^*^	14.79^*^	31.21^*^	8.56	11.82^*^	8.59
Dormitory	27	4	6	20	26	3	4	19
Rented accommodation	25	3	5	19	23	3	5	20
Living with relatives	26	4	5	20	28	3	5	19
Living with a friend	21.5	4	5	20	22.5	2.5	4.5	17
At home	22	3	4	19	22.5	2.5	4	19
**Distance between home and college** (Diff.)	2.51	1.53	1.81	0.20	4.43	4.09	9.57^*^	3.00
≤20.0	25	3	5	19	23	3	4	19
20.1–50.0	24	3	5	20	24	3	4	19
50.1–100.0	24	4	5	19	24.5	3	4	19
≥100.1	23	3	5	20	25	3	5	19

*CZ, Czech Republic; SK, Slovak Republic; IAT, Internet Addiction Test; GAD-7, Generalized Anxiety Disorder instrument; PHQ-9, Patient Health Questionnaire for depressive symptoms; PSS, Perceived Stress Scale; Diff., difference*.

* *significant difference at α < 0.05*.

Regarding the results of the difference tests, significant differences between countries were identified in almost all mental health indicators. The only exception was Internet addiction as measured by the IAT [Mann-Whitney *U*-test statistic (*p*-value): IAT = 976,891.0 (0.137); GAD-7 = 949,355.0 (0.006); PHQ-9 = 951,687.0 (0.008); PSS = 954,841.0 (0.012)]. Table 2 also presents an analysis of the differences in median scores between the individual categories of students. For comparison purposes, this analysis included the median scores reported by students categorized by age, gender, and study-related characteristics. In most categories, differences were confirmed at the significance level of α < 0.05. The lowest number of significant differences was found in the categories related to the study degree and distance between college and home. Overall, significant differences in the IAT, GAD-7, PHQ-9, and PSS scores were found between categories related to age, gender, and study-related characteristics in the majority of the analyzed cases.

Table 3 provides a closer look at the prevalence of mental health problems among Czech and Slovak students during the COVID-19 pandemic. In this context, the focus was on mild (moderate in the case of PSS) and higher levels of severity of mental health problems. On this basis, 27.2% of Czech students met the criteria for mild Internet addiction, 3.4% for moderate Internet addiction, and 0.1% for severe Internet addiction. In the case of Slovak students, these severity ranges were 27, 6, and 0.2%, respectively. The prevalence of mild, moderate, and severe anxiety symptoms among Czech students were 26.2, 9.2, and 4.9%, respectively. In Slovak Republic, mild, moderate, and severe anxiety symptoms occurred in 23, 8.1, and 3.5% of students, respectively. Depressive symptoms among Czech students were as follows: 28.6% of students had mild symptoms, 13.6% had moderate symptoms, 6.4% had moderately severe symptoms, and 3.4% had severe symptoms. Slovak students reported depressive symptoms as follows: 28.6% of students had mild symptoms, 11% had moderate symptoms, 5.4% had moderately severe symptoms, and 2.7% had severe symptoms. The highest prevalence was found for stress. Moderate and high stresses were reported by 74.1 and 12.9% of Czech students, respectively. In Slovak Republic, 79.4% of students reported moderate and 9.1% high stress.

**Table 3:** Frequency of mental health problems in severity classification.

**IAT**		**GAD-7**		**PSS**		**PHQ-9**	
**Range**	***n*** **(%)**	**Range**	***n*** **(%)**	**Range**	***n*** **(%)**	**Range**	***n*** **(%)**
**CZ**
No	984 (69.2%)	No	849 (59.7%)	Low	186 (13.1%)	No	683 (48%)
Mild	387 (27.2%)	Mild	372 (26.2%)	Moderate	1,053 (74.1%)	Mild	406 (28.6%)
Moderate	49 (3.4%)	Moderate	131 (9.2%)	High	183 (12.9%)	Moderate	193 (13.6%)
Severe	2 (0.1%)	Severe	70 (4.9%)	–	–	Moderately severe	91 (6.4%)
–	–	–	–	–	–	Severe	49 (3.4%)
**SK**
No	1,122 (66.9%)	No	1,097 (65.4%)	Low	193 (11.5%)	No	877 (52.3%)
Mild	452 (27%)	Mild	385 (23%)	Moderate	1,331 (79.4%)	Mild	479 (28.6%)
Moderate	100 (6%)	Moderate	136 (8.1%)	High	153 (9.1%)	Moderate	185 (11%)
Severe	3 (0.2%)	Severe	59 (3.5%)	–	–	Moderately severe	90 (5.4%)
–	–	–	–	–	–	Severe	46 (2.7%)

*IAT, Internet Addiction Test; GAD-7, Generalized Anxiety Disorder instrument; PHQ-9, Patient Health Questionnaire for depressive symptoms; PSS, Perceived Stress Scale; n, frequency; CZ, Czech Republic; SK, Slovak Republic*.

The following quantile regression analysis was used to evaluate the associations between Internet addiction as an independent variable and anxiety symptoms, depressive symptoms, and stress as dependent (explanatory) variables. The analysis included total scores of IAT, GAD-7, PHQ-9, and PSS and was applied in three intervals of the dependent variables: λ = 0.25 students with the lowest rate of mental health problem, λ = 0.5 students with moderate rate of mental health problem, and λ = 0.75 students with the highest rate of mental health problem in terms of GAD-7, PHQ-9, or PSS.

**Figure 1:**
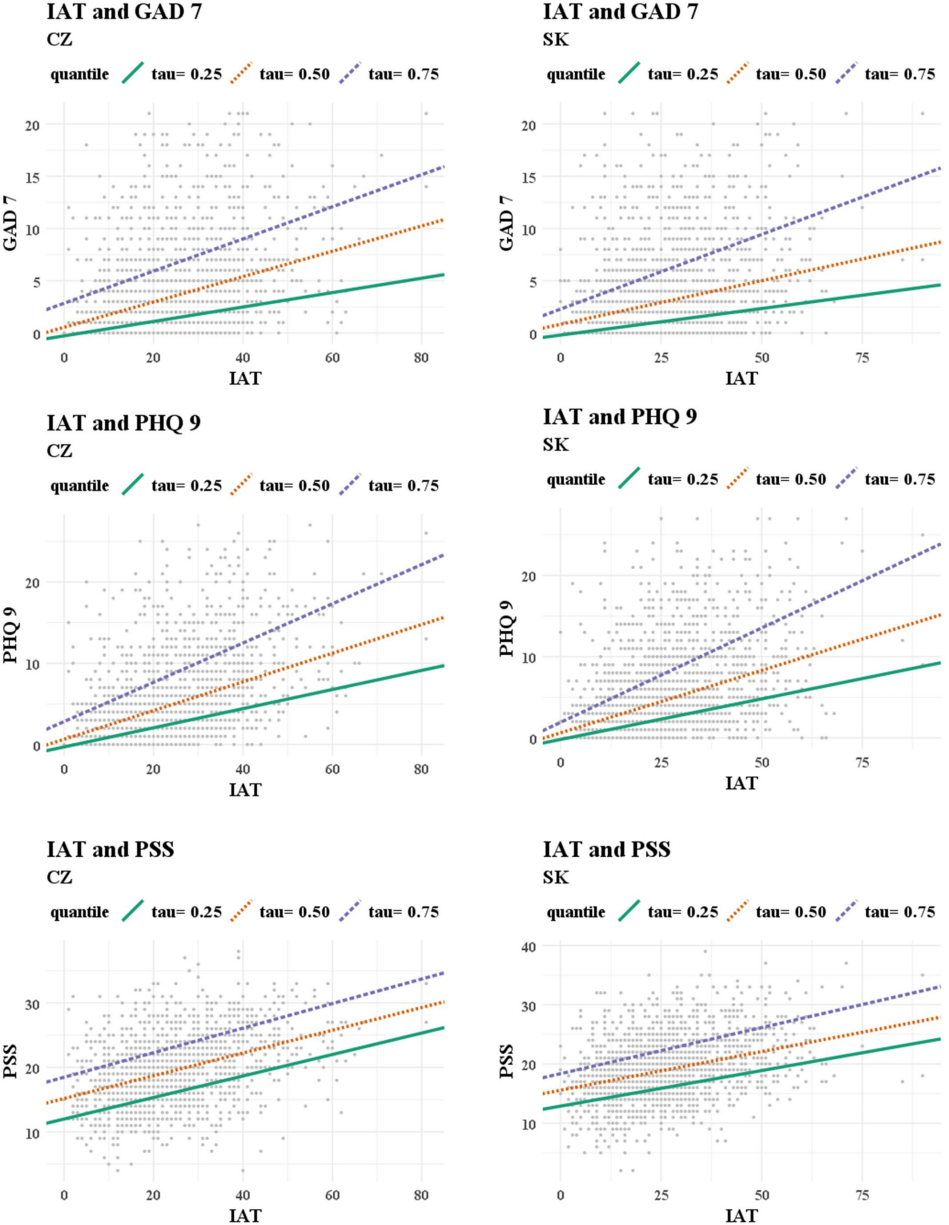
Visualization of the associations between Internet addiction and mental health problems (outcomes of regression models).

Table 4 provides the results for the stated hypotheses H1a, H1b, and H1c. Based on the results of the quantile regression analysis, it was possible to confirm the significant and positive associations between Internet addiction and anxiety symptoms, depressive symptoms, and stress in all of the analyzed cases. In other words, higher scores of IAT were associated with higher scores of GAD-7, PHQ-9, and PSS, regardless of the rate of these mental health problems. For a better understanding of the associations, a visualization (Figure 1) was created showing the individual observations as well as the linear curves corresponding to the outputs of the regression models. As noted above, a positive trend was evident in all of the analyzed cases.

**Table 4:** Results of quantile regression analysis—associations between Internet addiction as an independent variable and mental health problems as dependent (explanatory) variables.

	**CZ**			**SK**		
	**λ** **=** **0.25**	**λ** **=** **0.50**	**λ** **=** **0.75**	**λ** **=** **0.25**	**λ** **=** **0.50**	**λ** **=** **0.75**
	**Coef. (** ***p*** **-value)**	**Coef. (** ***p*** **-value)**	**Coef. (** ***p*** **-value)**	**Coef. (** ***p*** **-value)**	**Coef. (** ***p*** **-value)**	**Coef. (** ***p*** **-value)**
**H1a (IAT** ** → GAD-7)**
α	−0.276 (0.22)	0.545 (0.048)	2.846 (<0.001)	−0.203 (0.241)	0.833 (<0.001)	2.286 (<0.001)
β	0.069 (<0.001)	0.121 (<0.001)	0.154 (<0.001)	0.05 (<0.001)	0.083 (<0.001)	0.143 (<0.001)
*R* ^2^	0.041	0.056	0.057	0.021	0.042	0.065
**H1b (IAT** ** → PHQ-9)**
α	−0.294 (0.265)	0.647 (0.036)	2.828 (<0.001)	−2.0 (0.331)	0.615 (0.016)	1.907 (<0.001)
β	0.118 (<0.001)	0.176 (<0.001)	0.241 (<0.001)	0.10 (<0.001)	0.154 (<0.001)	0.233 (<0.001)
*R* ^2^	0.070	0.092	0.107	0.057	0.090	0.115
**H1c (IAT** ** → PSS)**
α	12.0 (<0.001)	15.18 (<0.001)	18.476 (<0.001)	12.88 (<0.001)	15.565 (<0.001)	18.356 (<0.001)
β	0.167 (<0.001)	0.176 (<0.001)	0.190 (<0.001)	0.12 (<0.001)	0.13 (<0.001)	0.165 (<0.001)
*R* ^2^	0.067	0.076	0.080	0.052	0.054	0.072

*CZ, Czech Republic; SK, Slovak Republic; Coef., coefficient; IAT, Internet Addiction Test; GAD-7, Generalized Anxiety Disorder instrument; PHQ-9, Patient Health Questionnaire for depressive symptoms; PSS, Perceived Stress Scale*.

The following logistic regression analyses were used to assess the relationships between selected study-related characteristics and mental health problems (IAT, GAD-7, PHQ-9, PSS) in their binomial form. For this purpose, the scores for Internet addiction, anxiety symptoms, depressive symptoms, and stress were adjusted as follows: the lowest possible range (“No” for IAT, GAD-7, PHQ-9; “Low” for PSS) was coded as 0, while the other ranges indicating an increased severity of mental health problems were coded as 1. Thus, in binomial form, 0 represented the case where students did not report an increased level of mental health problems, and 1 represented the case where students reported an increased level of mental health problems. The selected study-related characteristics are arranged in separate (Tables 5–**9**). The analysis was also conducted in the variant with the included control variable, which was gender with the reference category—female. A relationship whose significance was evident in both the model without the control variable and the model with the included control variable proved to be more reliable.

**Table 5:** Results of logistic regression analysis with the degree of study as an independent variable.

**Ref. cat.: 3rd degree**	**IAT**		**GAD-7**		**PHQ-9**		**PSS**	
	**β** **(Sig)**	**OR (95% CI)**	**β** **(Sig)**	**OR (95% CI)**	**β** **(Sig)**	**OR (95% CI)**	**β** **(Sig)**	**OR (95% CI)**
**CZ**
1st degree	0.001	1 (0.75–1.33)	−0.408^***^	0.67 (0.51–0.87)	−0.131	0.88 (0.67–1.14)	0.01	1.01 (0.68–1.5)
2nd degree	−0.196	0.82 (0.6–1.13)	−0.364^**^	0.7 (0.52–0.94)	−0.299^**^	0.74 (0.55–1)	−0.184	0.83 (0.54–1.28)
Combined 1st and 2nd	0.539^*^	1.71 (0.94–3.14)	−0.052	0.95 (0.52–1.72)	0.459	1.58 (0.85–2.95)	0.053	1.05 (0.42–2.63)
Nagelkerke *R^2^*	0.006		0.051		0.008		0.002	
**SK**
1st degree	0.409	1.51 (0.86–2.64)	−0.431^*^	0.65 (0.4–1.06)	−0.205	0.81 (0.5–1.33)	−0.034	0.97 (0.43–2.16)
2nd degree	0.388	1.47 (0.82–2.64)	−0.609^**^	0.54 (0.32–0.91)	−0.45^*^	0.64 (0.38–1.07)	−0.292	0.75 (0.33–1.71)
Combined 1st and 2nd	0.652	1.92 (0.83–4.42)	−0.59	0.55 (0.25–1.25)	−0.623	0.54 (0.24–1.18)	−0.748	0.47 (0.16–1.42)
Nagelkerke *R*^2^	0.002		0.011		0.006		0.005	
**Model with gender as a control variable (Ref. cat.: Female)**								
**CZ**
1st degree	0.006	1.01 (0.76–1.34)	−0.445^†^	0.64 (0.49–0.84)	−0.146	0.86 (0.66–1.13)	−0.033	0.97 (0.64–1.45)
2nd degree	−0.201	0.82 (0.59–1.13)	−0.353^**^	0.7 (0.52–0.95)	−0.289^*^	0.75 (0.56–1.01)	−0.151	0.86 (0.55–1.34)
Combined 1st and 2nd	0.541^*^	1.72 (0.94–3.14)	−0.064	0.94 (0.51–1.72)	0.458	1.58 (0.84–2.96)	0.038	1.04 (0.41–2.64)
CV – Gender (Male)	0.161	1.17 (0.91–1.52)	−0.89^†^	0.41 (0.31–0.54)	−0.475^†^	0.62 (0.49–0.79)	−1.258^†^	0.28 (0.21–0.39)
Nagelkerke *R*^2^	0.008		0.01		0.021		0.075	
**SK**
1st degree	0.463	1.59 (0.9–2.8)	−0.467^*^	0.63 (0.38–1.03)	−0.22	0.8 (0.49–1.31)	−0.15	0.86 (0.38–1.94)
2nd degree	0.435	1.54 (0.86–2.78)	−0.642^**^	0.53 (0.31–0.89)	−0.463^*^	0.63 (0.38–1.05)	−0.399	0.67 (0.29–1.55)
Combined 1st and 2nd	0.734^*^	2.08 (0.9–4.82)	−0.645	0.52 (0.23–1.19)	−0.647	0.52 (0.24–1.15)	−0.95^*^	0.39 (0.13–1.18)
CV – Gender (Male)	0.419^†^	1.52 (1.23–1.88)	−0.296^***^	0.74 (0.6–0.92)	−0.131	0.88 (0.72–1.07)	−0.958^†^	0.38 (0.28–0.52)
Nagelkerke *R^2^*	0.015		0.005		0.007		0.049	

*Significance*:

* *p-value < 0.1*,

** *p-value < 0.05*,

*** *p-value < 0.01*,

† *p-value < 0.001*.

*Ref. cat., reference category, IAT, Internet Addiction Test, GAD-7, Generalized Anxiety Disorder instrument, PHQ-9, Patient Health Questionnaire for depressive symptoms, PSS, Perceived Stress Scale, Sig, significance, OR, odds ratio, 95% CI, 95% confidence interval, CZ, Czech Republic, SK, Slovak Republic, CV, control variable*.

Table 5 focuses on H2a and shows the results of the logistic regression analysis, in which the study degree was considered an independent variable. In terms of Internet addiction (IAT), there was no significant relationship at the level of α < 0.05. However, a significant relationship at the level of α < 0.1 was observed for the combined first and second degrees. This was evident especially in Czech Republic, where significance was observed in both models. In this case, it was possible to state with caution that combined-degree students were more likely to report Internet addiction compared to third-degree students. With a focus on anxiety symptoms (GAD-7), significant relationships were identified for first- and second-degree studies in both countries. The negative β coefficients indicated a lower likelihood of anxiety symptoms in first- and second-degree students compared to third-degree students. Regarding depressive symptoms (PHQ-9), a significant relationship was confirmed for the second degree, especially in Czech Republic. Czech second-degree students were less likely to suffer from depressive symptoms than third-degree students. In Slovak Republic, the significance of this relationship was observed at the level of α < 0.1. In the case of stress (PSS), no significant relationship was found at the level of α < 0.05.

Table 6 focuses on H2b and presents the results of the logistic regression analysis, in which the form of study was used as an independent variable. Significant relationships were found in most of the analyzed cases. In these significant cases, the results showed that full-time students were more likely to have mental health problems such as Internet addiction (IAT), anxiety symptoms (GAD-7), depressive symptoms (PHQ-9), and stress (PSS) compared to part-time students. Closer and more significant relationships were especially in Czech Republic.

**Table 6:** Results of logistic regression analysis with the form of study as an independent variable.

**Ref. cat.: Part-time**	**IAT**		**GAD-7**		**PHQ-9**		**PSS**	
	**β** **(Sig)**	**OR (95% CI)**	**β** **(Sig)**	**OR (95% CI)**	**β** **(Sig)**	**OR (95% CI)**	**β** **(Sig)**	**OR (95% CI)**
**CZ**
Full-time	0.639^†^	1.9 (1.44–2.5)	0.234^*^	1.26 (0.99–1.61)	0.374^***^	1.45 (1.15–1.84)	0.475^***^	1.61 (1.16–2.23)
Nagelkerke *R*^2^	0.022		0.003		0.009		0.010	
**SK**
Full-time	0.458^**^	1.58 (1.04–2.4)	0.19	1.21 (0.82–1.79)	0.623^†^	1.86 (1.27–2.73)	0.462^*^	1.59 (0.97–2.6)
Nagelkerke *R*^2^	0.004		0.001		0.009		0.004	
**Model with gender as a control variable (Ref. cat.: Female)**								
**CZ**
Full-time	0.632^†^	1.88 (1.43–2.48)	0.295^**^	1.34 (1.05–1.72)	0.41^†^	1.51 (1.19–1.91)	0.605^†^	1.83 (1.31–2.57)
CV – Gender (Male)	0.112	1.12 (0.86–1.45)	−0.898^†^	0.41 (0.31–0.53)	−0.51^†^	0.6 (0.47–0.77)	−1.321^†^	0.27 (0.19–0.37)
Nagelkerke *R*^2^	0.022		0.046		0.025		0.089	
**SK**
Full-time	0.488^**^	1.63 (1.07–2.48)	0.174	1.19 (0.8–1.76)	0.617^***^	1.85 (1.27–2.71)	0.414	1.51 (0.92–2.5)
CV – Gender (Male)	0.418^†^	1.52 (1.23–1.87)	−0.282^***^	0.75 (0.61–0.93)	−0.115	0.89 (0.73–1.09)	−0.94^†^	0.39 (0.29–0.53)
Nagelkerke *R*^2^	0.017		0.006		0.010		0.046	

*Significance*:

* *p-value < 0.1*,

** *p-value < 0.05*,

*** *p-value < 0.01*,

† *p-value < 0.001*.

*Ref. cat., reference category; IAT, Internet Addiction Test; GAD-7, Generalized Anxiety Disorder instrument; PHQ-9, Patient Health Questionnaire for depressive symptoms; PSS, Perceived Stress Scale; Sig, significance; OR, odds ratio; 95% CI, 95% confidence interval; CZ, Czech Republic; SK, Slovak Republic; CV, control variable*.

The results of the logistic regression analysis with the field of study as an independent variable are shown in Table 7, which focuses on H2c and evidently provides many diverse results. The study field of Informatics, Mathematics, and Information and Communication Technologies (ICT) was used as the reference category in the regression models. For Internet addiction as measured by the IAT, there were several significant relationships with a negative β coefficient. Based on these results, a lower likelihood of Internet addiction was observed in students of study fields other than Informatics, Mathematics, and ICT. In Czech Republic, they were students of Education, Humanities and Arts; Social, Economic, and Legal Sciences; Design, Technology, Production, and Communications; Health Service, but also Services (tourism, sports, security, transport, logistics). Regarding anxiety symptoms (GAD-7), some significant results were confirmed with apparent discrepancies between countries. Significant relationships with a positive β coefficient were observed especially in Czech students of individual study fields such as Education, Agricultural and Veterinary Sciences, and Health Service. In other words, Czech students of these study fields were more likely to be anxious than students of Informatics, Mathematics, and ICT. Significant relationships with a negative β coefficient were observed especially in Slovak Republic. In this respect, especially Slovak students of Natural Science were less likely to have anxiety symptoms than students of Informatics, Mathematics, and ICT. Several significant results were also found for depressive symptoms (PHQ-9), with countries showing some inconsistencies. Slovak Republic was characterized by a higher number of significant relationships with negative β coefficients. Thus, in particular, Slovak students of study fields other than Informatics, Mathematics, and ICT were less prone to depressive symptoms. With a focus on perceived stress as measured by the PSS, the results pointed in particular to the fact that Czech students of Design, Technology, Production, and Communications were less likely to be stressed compared to students of Informatics, Mathematics, and ICT. At the same time, it was possible to note that Slovak students of Humanities and Arts had a higher likelihood of stress than students of Informatics, Mathematics, and ICT.

**Table 7:** Results of logistic regression analysis with the field of study as an independent variable.

**Ref. cat.: Informatics, Mathematics & ICT**	**IAT**		**GAD-7**		**PHQ-9**		**PSS**	
	**β** **(Sig)**	**OR (95% CI)**	**β** **(Sig)**	**OR (95% CI)**	**β** **(Sig)**	**OR (95% CI)**	**β** **(Sig)**	**OR (95% CI)**
**CZ**
Education	−0.764^**^	0.47 (0.25–0.88)	0.991^***^	2.69 (1.34–5.42)	0.213	1.24 (0.66–2.32)	0.121	1.13 (0.41–3.1)
Humanities & Arts	−0.909^**^	0.4 (0.2–0.83)	0.536	1.71 (0.79–3.7)	−0.393	0.67 (0.34–1.36)	−0.434	0.65 (0.22–1.89)
Social, Economic & Legal Sciences	−0.887^***^	0.41 (0.23–0.75)	0.57^*^	1.78 (0.91–3.51)	−0.201	0.82 (0.45–1.49)	−0.143	0.87 (0.33–2.26)
Natural Science	−0.663	0.52 (0.23–1.17)	0.719	2.05 (0.86–4.87)	−0.254	0.78 (0.35–1.73)	0.338	1.4 (0.35–5.58)
Design, Technology, Production & Communications	−1.001^***^	0.37 (0.18–0.77)	–−0.015	0.99 (0.44–2.21)	−0.369	0.69 (0.34–1.41)	−1.362^***^	0.26 (0.09–0.71)
Agricultural & Veterinary	−0.27	0.76 (0.36–1.62)	1.071^***^	2.92 (1.29–6.59)	0.474	1.61 (0.75–3.47)	−0.106	0.9 (0.27–2.95)
Health Service	−1.149^***^	0.32 (0.14–0.74)	1.116^***^	3.05 (1.31–7.12)	0.278	1.32 (0.59–2.93)	−0.025	0.98 (0.28–3.43)
Services	−0.896^**^	0.41 (0.19–0.89)	0.413	1.51 (0.66–3.44)	−0.261	0.77 (0.36–1.63)	−0.073	0.93 (0.28–3.04)
Nagelkerke *R*^2^	0.015	0.027	0.015	0.037
SK
Education	−0.618^**^	0.54 (0.3–0.98)	0.272	1.31 (0.75–2.29)	−0.401	0.67 (0.38–1.16)	0.125	1.13 (0.53–2.42)
Humanities & Arts	−0.173	0.84 (0.48–1.49)	0.002	1 (0.57–1.77)	0.065	1.07 (0.61–1.88)	1.609^**^	5 (1.45–17.24)
Social, Economic & Legal Sciences	−0.421^**^	0.66 (0.45–0.96)	−0.307	0.74 (0.5–1.08)	−0.6^***^	0.55 (0.38–0.8)	0.419	1.52 (0.92–2.52)
Natural Science	−0.362	0.7 (0.38–1.26)	−0.645^**^	0.52 (0.28–0.99)	−0.544*	0.58 (0.33–1.03)	0.231	1.26 (0.56–2.81)
Design, Technology, Production & Communications	−0.252	0.78 (0.49–1.24)	−0.104	0.9 (0.56–1.44)	−0.351	0.7 (0.45–1.11)	0.204	1.23 (0.65–2.3)
Agricultural & Veterinary	−0.149	0.86 (0.45–1.65)	0.129	1.14 (0.6–2.17)	−0.162	0.85 (0.45–1.61)	1.204*	3.33 (0.96–11.61)
Health Service	−0.604^**^	0.55 (0.34–0.87)	0.09	1.09 (0.69–1.72)	−0.351	0.7 (0.45–1.1)	0.528	1.69 (0.88–3.26)
Services	−0.286	0.75 (0.49–1.16)	−0.259	0.77 (0.5–1.19)	−0.603^***^	0.55 (0.36–0.84)	0.684^**^	1.98 (1.06–3.71)
Nagelkerke *R*^2^	0.008		0.013		0.019		0.017	
**Model with gender as a control variable (Ref. cat.: Female)**								
**CZ**
Education	−0.691^**^	0.5 (0.26–0.95)	0.671*	1.96 (0.96–4.01)	0.005	1 (0.53–1.91)	−0.461	0.63 (0.22–1.78)
Humanities & Arts	−0.849^**^	0.43 (0.21–0.89)	0.263	1.3 (0.59–2.86)	−0.572	0.56 (0.28–1.15)	−0.922*	0.4 (0.13–1.19)
Social, Economic & Legal Sciences	−0.826^***^	0.44 (0.24–0.81)	0.307	1.36 (0.68–2.72)	−0.379	0.68 (0.37–1.26)	−0.619	0.54 (0.2–1.43)
Natural Science	−0.598	0.55 (0.24–1.26)	0.429	1.54 (0.64–3.71)	−0.445	0.64 (0.28–1.45)	−0.165	0.85 (0.21–3.46)
Design, Technology, Production & Communications	−1.022^***^	0.36 (0.17–0.75)	0.086	1.09 (0.48–2.47)	−0.316	0.73 (0.36–1.49)	−1.285^**^	0.28 (0.1–0.78)
Agricultural & Veterinary	−0.199	0.82 (0.38–1.76)	0.765*	2.15 (0.94–4.93)	0.275	1.32 (0.6–2.87)	−0.67	0.51 (0.15–1.72)
Health Service	−1.087^**^	0.34 (0.14–0.79)	0.852*	2.35 (0.99–5.56)	0.103	1.11 (0.49–2.49)	−0.503	0.6 (0.17–2.18)
Services	−0.873^**^	0.42 (0.19–0.91)	0.306	1.36 (0.59–3.14)	−0.335	0.72 (0.34–1.52)	−0.244	0.78 (0.24–2.61)
CV – Gender (Male)	0.163	1.18 (0.89–1.55)	−0.771^†^	0.46 (0.35–0.62)	−0.458^†^	0.63 (0.49–0.82)	−1.175^†^	0.31 (0.22–0.44)
Nagelkerke *R*^2^	0.016		0.054		0.030		0.091	
**SK**
Education	−0.465	0.63 (0.34–1.15)	0.132	1.14 (0.65–2.01)	−0.491*	0.61 (0.35–1.07)	−0.271	0.76 (0.35–1.67)
Humanities & Arts	0.015	1.02 (0.57–1.82)	−0.167	0.85 (0.47–1.52)	−0.042	0.96 (0.54–1.71)	1.142*	3.13 (0.89–10.97)
Social, Economic & Legal Sciences	−0.278	0.76 (0.51–1.11)	−0.44^**^	0.64 (0.44–0.95)	−0.684^†^	0.5 (0.34–0.74)	0.064	1.07 (0.63–1.8)
Natural Science	−0.249	0.78 (0.43–1.42)	−0.753^**^	0.47 (0.25–0.89)	−0.61^**^	0.54 (0.3–0.97)	−0.047	0.95 (0.42–2.16)
Design, Technology, Production & Communications	−0.295	0.74 (0.47–1.19)	−0.065	0.94 (0.59–1.5)	−0.328	0.72 (0.46–1.14)	0.298	1.35 (0.71–2.54)
Agricultural & Veterinary	−0.001	1 (0.52–1.93)	−0.005	1 (0.52–1.91)	−0.247	0.78 (0.41–1.49)	0.853	2.35 (0.66–8.29)
Health Service	−0.406	0.67 (0.41–1.08)	−0.089	0.91 (0.57–1.47)	−0.465^**^	0.63 (0.4–1)	0.01	1.01 (0.51-2)
Services	−0.165	0.85 (0.55–1.31)	−0.372	0.69 (0.44–1.08)	−0.674^***^	0.51 (0.33–0.78)	0.397	1.49 (0.78–2.82)
CV – Gender (Male)	0.38^***^	1.46 (1.16–1.83)	−0.344^***^	0.71 (0.56–0.9)	−0.214*	0.81 (0.65–1)	−0.963^†^	0.38 (0.27–0.53)
Nagelkerke *R*^2^	0.017		0.020		0.018		0.055	

*Significance*:

* *p-value < 0.1*,

** *p-value < 0.05*,

*** *p-value < 0.01*,

† *p-value < 0.001*.

*Ref. cat., reference category, IAT, Internet Addiction Test, GAD-7, Generalized Anxiety Disorder instrument, PHQ-9, Patient Health Questionnaire for depressive symptoms, PSS, Perceived Stress Scale, Sig, significance, OR, odds ratio, 95% CI, 95% confidence interval, CZ, Czech Republic, SK, Slovak Republic, CV, control variable*.

Table 8 focuses on H2d and shows the results of the logistic regression analysis, in which housing during the semester was an independent variable in its binomial form [Away from home (dormitory, rented accommodation, living with relatives, living with a friend) compared to Home]. In terms of Internet addiction (IAT) and depressive symptoms (PHQ-9), significant relationships with a positive β coefficient were confirmed in all of the analyzed cases. In both countries, the results clearly indicated that students who lived away from home during the semester were more likely to suffer from Internet addiction and depressive symptoms than students who lived at home. In the case of anxiety symptoms (GAD-7), a significant relationship was revealed only in Slovak Republic. In this context, being a student living away from home during the semester increased the likelihood of anxiety symptoms during the COVID-19 pandemic. Focusing on perceived stress (PSS), no significant relationship was found at the level of α < 0.05. The only relationship was observed at the significance level of α < 0.1 in Slovak Republic.

**Table 8:** Results of logistic regression analysis with housing during the semester as an independent variable.

**Ref. cat.: Home**	**IAT**		**GAD-7**		**PHQ-9**		**PSS**	
	**β** **(Sig)**	**OR (95% CI)**	**β** **(Sig)**	**OR (95% CI)**	**β** **(Sig)**	**OR (95% CI)**	**β** **(Sig)**	**OR (95% CI)**
**CZ**
Away from home	0.434^†^	1.54 (1.23–1.94)	0.164	1.18 (0.95–1.46)	0.236^**^	1.27 (1.03–1.56)	0.124	1.13 (0.83–1.54)
Nagelkerke *R*^2^	0.014		0.002		0.005		0.001	
**SK**
Away from home	0.458^†^	1.58 (1.28–1.95)	0.25^**^	1.28 (1.05–1.58)	0.195^**^	1.22 (1–1.47)	0.284^*^	1.33 (0.98–1.79)
Nagelkerke *R*^2^	0.015	0.005	0.003	0.004
**Model with gender as a control variable (Ref. cat.: Female)**								
**CZ**
Away from home	0.433^†^	1.54 (1.23–1.94)	0.178	1.19 (0.96–1.48)	0.244^**^	1.28 (1.03–1.57)	0.145	1.16 (0.84–1.59)
CV – Gender (Male)	0.145	1.16 (0.89–1.5)	−0.88^†^	0.41 (0.32–0.54)	−0.484^†^	0.62 (0.48–0.79)	−1.266^†^	0.28 (0.21–0.39)
Nagelkerke *R*^2^	0.015		0.043		0.019		0.075	
**SK**
Away from home	0.466^†^	1.59 (1.29–1.97)	0.248^**^	1.28 (1.04–1.57)	0.194^**^	1.21 (1–1.47)	0.28^*^	1.32 (0.98–1.79)
CV – Gender (Male)	0.418^†^	1.52 (1.23–1.87)	−0.283^***^	0.75 (0.61–0.93)	−0.123	0.88 (0.72–1.08)	−0.945^†^	0.39 (0.29–0.53)
Nagelkerke *R*^2^	0.028		0.010		0.004		0.047	

*Significance*:

* *p-value < 0.1*,

** *p-value < 0.05*,

*** *p-value < 0.01*,

† *p-value < 0.001*.

*Ref. cat., reference category, IAT, Internet Addiction Test, GAD-7, Generalized Anxiety Disorder instrument, PHQ-9, Patient Health Questionnaire for depressive symptoms, PSS, Perceived Stress Scale, Sig, significance, OR, odds ratio, 95% CI, 95% confidence interval, CZ, Czech Republic, SK, Slovak Republic, CV, control variable*.

Table 9 focuses on H2e and presents the results of the logistic regression analysis, in which the distance between home and college was considered an independent variable. It is clear that most of the analyzed cases were not significant. Specifically, only two significant relationships were found in Slovak Republic. In the first case, Slovak students who traveled between 50.1 and 100 km from home to college were less prone to suffer from depressive symptoms than students who traveled more than 100 km. In the second case, Slovak students who traveled 20 km or less from home to college had a lower likelihood of stress than students traveling more than 100 km.

**Table 9:** Results of logistic regression analysis with distance between home and college as an independent variable.

**Ref. cat.: ≥100.1**	**IAT**		**GAD-7**		**PHQ-9**		**PSS**	
	**β** **(Sig)**	**OR (95% CI)**	**β** **(Sig)**	**OR (95% CI)**	**β** **(Sig)**	**OR (95% CI)**	**β** **(Sig)**	**OR (95% CI)**
**CZ**
≤20.0	0.134	1.14 (0.83–1.57)	−0.074	0.93 (0.69–1.25)	−0.137	0.87 (0.65–1.17)	0.061	1.06 (0.69–1.64)
20.1–50.0	0.089	1.09 (0.77–1.54)	0.054	1.06 (0.77–1.46)	−0.112	0.89 (0.65–1.23)	−0.022	0.98 (0.61–1.56)
50.1–100.0	0.04	1.04 (0.74–1.46)	−0.041	0.96 (0.7–1.32)	−0.006	0.99 (0.73–1.36)	0.007	1.01 (0.64–1.59)
Nagelkerke *R*^2^	0.001		0.001		0.001		<0.001	
**SK**
≤20.0	−0.2	0.82 (0.62–1.09)	−0.232	0.79 (0.6–1.05)	−0.064	0.94 (0.72–1.22)	−0.478^**^	0.62 (0.41–0.93)
20.1–50.0	−0.135	0.87 (0.65–1.17)	−0.098	0.91 (0.68–1.21)	−0.055	0.95 (0.72–1.24)	−0.134	0.87 (0.56–1.37)
50.1–100.0	0.032	1.03 (0.79–1.36)	−0.099	0.91 (0.69–1.19)	−0.27^**^	0.76 (0.59–0.99)	−0.174	0.84 (0.55–1.29)
Nagelkerke *R*^2^	0.003	0.002	0.004	0.007
**Model with gender as a control variable (Ref. cat.: Female)**								
**CZ**
≤20.0	0.124	1.13 (0.82–1.56)	−0.017	0.98 (0.73–1.33)	−0.104	0.9 (0.67–1.21)	0.175	1.19 (0.76–1.86)
20.1–50.0	0.086	1.09 (0.77–1.54)	0.074	1.08 (0.78–1.49)	−0.103	0.9 (0.66–1.24)	0.012	1.01 (0.63–1.63)
50.1–100.0	0.036	1.04 (0.74–1.46)	−0.02	0.98 (0.71–1.35)	0.006	1.01 (0.74–1.38)	0.051	1.05 (0.66–1.68)
CV – Gender (Male)	0.143	1.15 (0.89–1.49)	−0.874^†^	0.42 (0.32–0.55)	−0.474^†^	0.62 (0.49–0.79)	−1.274^†^	0.28 (0.2–0.38)
Nagelkerke *R*^2^	0.002		0.041		0.015		0.075	
**SK**
≤20.0	−0.211	0.81 (0.61–1.08)	−0.227	0.8 (0.6–1.06)	−0.062	0.94 (0.72–1.23)	−0.469^**^	0.63 (0.41–0.95)
20.1–50.0	−0.142	0.87 (0.65–1.16)	−0.094	0.91 (0.68–1.21)	−0.053	0.95 (0.72–1.25)	−0.121	0.89 (0.56–1.39)
50.1–100.0	0.036	1.04 (0.79–1.36)	−0.101	0.9 (0.69–1.19)	−0.271^**^	0.76 (0.59–0.99)	−0.186	0.83 (0.54–1.28)
CV – Gender (Male)	0.41^†^	1.51 (1.22-1.86)	–−0.275^**^	0.76 (0.61–0.94)	−0.116	0.89 (0.73–1.09)	−0.949^†^	0.39 (0.29–0.53)
Nagelkerke *R*^2^	0.015		0.007		0.005	0.050

*Significance*:

** *p-value < 0.05*,

† *p-value < 0.001*.

*Ref. cat., reference category, IAT, Internet Addiction Test, GAD-7, Generalized Anxiety Disorder instrument, PHQ-9, Patient Health Questionnaire for depressive symptoms, PSS, Perceived Stress Scale, Sig, significance, OR, odds ratio, 95% CI, 95% confidence interval, CZ, Czech Republic, SK, Slovak Republic, CV, control variable*.

**Figure 2:**
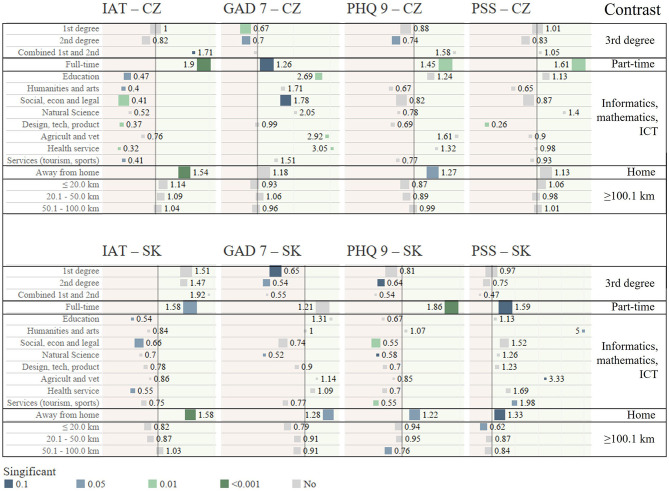
Visualization of odds ratios—relationships between selected study-related characteristics and mental health problems (IAT, GAD-7, PHQ-9, PSS).

Figure 2 visualizes the outputs of the logistic regression analyses presented in Tables 5–9. The odds ratios of the relationships from the regression models without the control variable are shown separately for both Czech Republic and Slovak Republic. The reference categories (Contrast) are displayed on the right side of the visualization. The size of the squares represents the frequency of observations in each category. The gray squares represent relationships that were not significant even at the level of α < 0.1. The values in the visualization represent odds ratios, and the further to the right the value is from 1 (vertical line), the higher the likelihood of a mental health problem compared to the reference category. Conversely, the further to the left is the value from 1 (vertical line), the lower the likelihood of a mental health problem compared to the reference category.

## Discussion

### Internet Addiction, Anxiety Symptoms, Depressive Symptoms, and Stress Among Students

Czech and Slovak students showed significant differences in scores for anxiety symptoms, depressive symptoms, and stress. In this respect, Czech students reported higher mean scores indicating their more frequent mental health problems. This means that, despite shared social priorities and a common history in which these countries formed a single entity, each country behaves as a unique living organism and is characterized by its own social specificities. The importance of cross-cultural differences when examining the mental health of students from more than one country was also highlighted by Ochnik et al. (69). Additionally, this study revealed differences between most of the study-specific categories. All these results could be explained by the different measures implemented in the countries during the pandemic.

The overall prevalence of Internet addiction (IAT > 30) was 30.7% among Czech and 33.2% among Slovak students during the first wave of the COVID-19 pandemic. The most serious levels of Internet addiction requiring increased attention were identified in 3.5% (moderate: 3.4%, severe: .1%) and 6.2% (moderate: 6%, severe: .2%) of Czech and Slovak students, respectively. Zhu et al. (33) found a similar prevalence and revealed that 28.4% of Chinese students developed Internet addiction, which correlated with their academic burnout. In view of these results, the cut-off point for IAT was ≥ 50. Using the same cut-off point, a similar prevalence (5.8%) was found in students from Thailand (70), while a higher prevalence was observed in American students (16.8%) (31), Spanish students (12.4%) (71), Iraqi students (23%) (32), Bangladeshi students (32.6%) (72), Chinese students (28.4%) (73), Indian students (10.1%) (47), as well as Brazil students (20%) (74). Interestingly, Khazaie et al. (75) found up to 68.8% prevalence of Internet addiction among Iranian medical students. The mean IAT scores indicated a normal level of Internet usage in both countries examined in this study. In comparison, similar scores were reported by students from Turkey (76), while higher scores indicating a mild Internet addiction were found in students from Iran (41), Japan (77), and Australia (78). Overall, it was possible to conclude that Czech and Slovak college students reported less Internet addiction than students from other countries. A possible explanation is cultural differences, which were highlighted in the study by Lozano-Blasco et al. (26).

In Czech Republic, 40.3% of students suffered from anxiety symptoms (GAD-7 ≥ 5), of whom 26.2% met the criteria for mild anxiety symptoms, 9.2% for moderate anxiety symptoms, and 4.9% for severe anxiety symptoms. For Slovak Republic, anxiety symptoms occurred in 34.6% of students, with 23, 8.1, and 3.5% of students reporting mild, moderate, and severe anxiety symptoms, respectively. Using the standard cut-off point of GAD-7 ≥ 10, it was possible to identify 14.1% of Czech and 11.6% of Slovak students with anxiety symptoms requiring increased attention. Interestingly, Hajduk et al. (57) used the same cut-off point and found a considerable higher prevalence of anxiety symptoms among Slovak higher education students (43.3%). This can be explained by the fact that the research in this study was conducted during the first wave, when the situation in the country was not so critical. On the contrary, Hajduk et al. (57) conducted their research at the end of 2020, when the situation was much more serious and this may have been reflected in the mental health outcomes. A higher prevalence was also found in Saudi medical students (32.4%) (79), Pakistani medical students (30.3%) (80), Portuguese students (23.9%) (81), Polish students (38.4%) (82), Turkish students (44.5%) (83), and Brazilian students (53.8%) (84). The mean GAD-7 score was 4.71 for Czech students and 4.15 for Slovak students. Czech students reported a similar mean score also in the study conducted by Ochnik et al. (69) during the first wave of the COVID-19 pandemic. Compared to the study examining nine countries (38), higher mean scores were reported by students from Poland (9.2), Slovenia (7.37), Russia (7.48), Turkey (10.41), Israel (7.92), Colombia (8.45), and Ukraine (6.15), while only students from Germany reported a lower mean score (2.92). The mean GAD-7 score of Czech students was consistent with this study. A slightly higher mean score was found in China (5.38) (85). It is clear that students from Czech Republic and Slovak Republic suffered from anxiety symptoms during the COVID-19 pandemic to a lesser extent than students from other countries.

During the COVID-19 pandemic, depressive symptoms were prevalent in 52% of Czech students and 47.7% of Slovak students. More specifically, mild depressive symptoms occurred in the same proportion of Czech and Slovak students, i.e., in 28.6%. Moderate depressive symptoms were reported by 13.6% of Czech and 11% of Slovak students, while moderately severe depressive symptoms were reported by 6.4% of Czech and 5.4% of Slovak students. The most severe depressive symptoms were identified in a not negligible proportion of students, i.e., in 3.4% of Czech students and 2.7% of Slovak students. In terms of the cut-off point of PHQ-9 ≥ 10, 23.4% of Czech students and 19.1% of Slovak students had depressive symptoms, which needs to be addressed. As seen in anxiety symptoms, this study revealed a lower prevalence of depressive symptoms among Slovak students than the study by Hajduk et al. (57), who conducted their research at the peak of the second wave of the pandemic, when the situation was more dramatic than in the first wave. Slovak students also reported more depression than anxiety, which is in line with the results revealed by Hajduk et al. (57). In comparison, a similar prevalence was observed among Indian doctoral students (26.7%) (86), Chinese students (15.8%) (87), and Korean students (14%) (88), while a higher prevalence was observed among students from Germany (37%) (89), Bangladesh (48.8%) (90), and Malaysia (33.8%) (91). The mean score was 6.34 for Czech students and 5.77 for Slovak students, indicating mild depressive symptoms. Based on the mean score, mild depressive symptoms were also reported by German (6.77) and Chinese (6.99) students (85). A higher mean score reported Bangladeshi students (9.5) (90), Polish students (11.3) (92), and Greek students (9.36) (93).

In Czech Republic, 74.1 and 12.9% of students reported moderate and high stress, respectively. In Slovak Republic, 79.4% of students reported moderate and 9.1% high stress. Using the cut-off point of PSS ≥ 27, a higher prevalence of stress showed students from India (31.5%) (94) and Saudi Arabia (19.6%) (79). On the other hand, a similar prevalence was found in Sri Lanka (11.8%) (95) and Polish students (43%) (96). The mean PSS score was 19.82 in Czech Republic and 19.28 in Slovak Republic. The perception of stress by Slovak students in this study was in line with the study by Rutkowska et al. (61). A slightly higher mean score was reported by French students (21.9) (97). When comparing the results of this study with those of the study on the mental health of students from nine countries (38), similar mean scores were found in Slovenia (19.83), Ukraine (19.93), Israel (21.51), and Colombia (21.37), while slightly higher mean scores were found in Turkey (22.71), Poland (22.69), Germany (22.54), and Russia (21.98). Czech students' perception of stress was consistent with this study (18.16).

### Associations of Internet Addiction With Anxiety Symptoms, Depressive Symptoms, and Stress

The quantile regression analysis used in this study revealed the significant positive associations of Internet addiction with anxiety symptoms, depressive symptoms, and stress. These findings indicated that higher scores of IAT were associated with higher scores of GAD-7, PHQ-9, and PSS, regardless of the rate of the mental health problems. The stated hypotheses H1a, H1b, and H1c were supported. On this basis, it was possible to agree that Internet addiction is associated with other mental health problems in young people, pointing to a threatening element in their lives. Similar findings can be found in previous studies from other countries (28, 29, 31, 32, 98). In this context, problematic Internet use may be a predictor in screening high-risk students for mental health problems (30). Jiang et al. (27) also revealed that internet addiction was directly related to college students' depression and indirectly predicted students' depression *via* the mediator of social support and sleep quality. On the other hand, under the mediation of fear of missing out, young people with anxiety are more likely to develop Internet gaming disorder, while young people with depression or stress may be susceptible to other types of Internet use disorders (99). In this context, Yang et al. (100) confirmed that baseline Internet addiction had a significant net-predictive effect on follow-up depression, but it was also true that baseline depression had a significant net-predictive effect on follow-up Internet addiction. Thus, the issue can also be considered from the opposite perspective, in which mental health problems such as anxiety, depression, and stress are associated with Internet addiction in students (47, 72–74, 77).

The co-occurrence of Internet addiction and anxiety symptoms, depressive symptoms, and stress in students suggests that Internet addiction is positively associated with other mental health problems (41). The results could be explained by the ongoing pandemic and by the fact that excessive use of the Internet impairs psychological wellbeing, which can be a driver of psychological distress (27–31). Particularly in the context of the COVID-19 pandemic, the Internet has been used as an effective tool to provide distance education. In this way, the pandemic may have raised concerns about the mental health of young adults and contributed to the development of problematic Internet use by students, which may have further undesirable consequences (101). The Internet can provide a short-term reward during difficult times, but it can also lead to other behavioral addictions (102), with adolescence and young adulthood being the most vulnerable period of life (103). It is important to note that although digital technologies have dramatically changed the environment in which college students connect with each other, gain knowledge, and have fun, they also appear to have some detrimental effects on mental health (24). In addition, these problems adversely affect both academic performance and quality of life and lead to many other social and health consequences (48–53, 55, 56, 104–106). For these reasons, digital technologies should be used with caution, the higher education environment should adapt to this situation (107), and students at risk of Internet addiction should also be monitored for other psychological problems.

### Study-Related Predictors of Mental Health Problems

This study revealed that first- and second-degree students from Czech Republic and Slovak Republic were less likely to suffer from anxiety symptoms than third-degree students. In addition, especially Czech second-degree students were less prone to depressive symptoms compared to third-degree students. This could be due to the fact that the third degree of study is considered to be very demanding and challenging for students. Finally, it was found with caution that mainly Czech students of the combined study were more likely to be addicted to the Internet compared to third-degree students. Overall, it can be concluded that the degree of study is a factor that needs increased attention, especially in terms of anxiety. In view of the results, there is a need to monitor third-degree students, as emphasized by Kowalczyk et al. (37). Other studies have also shown that the study degree is an important factor in examining mental health problems among students (38, 39).

This study also contributes to the knowledge that full-time study can be considered a risk factor for students' mental health, as indicated in the studies conducted by Aristovnik et al. (39) or Stallman (40). This fact was observed among both Czech and Slovak students. More specifically, Czech full-time students were more likely to have Internet addiction, anxiety symptoms, depressive symptoms, and stress compared to part-time students, while Slovak full-time students were more likely to have Internet addiction and depressive symptoms than part-time students. A possible explanation may be that full-time students were more vulnerable in terms of the COVID-19 pandemic (108). Also, full-time students are generally under more pressure in the higher education environment. However, the opposite result was found by Esmaeelzadeh et al. (109), in which part-time students were considered a risk group. In any case, the findings of this study can be followed up with further insights into the form of study and mental health in the college environment.

With a focus on the field of study, most of the results indicated that students of Informatics, Mathematics, and ICT were more prone to mental health problems than students of other fields. These findings are indicative of the fact that the field of study should not be underestimated and overlooked when examining students' psychological problems. This has also been demonstrated in other studies, the results of which differ from those of this study (3, 41). For instance, in the study by Lipson et al. (42), students of Humanities and Art and Design were at higher risk for mental health problems, while in the study by Odriozola-González et al. (43), students of Humanities and Arts and Social Sciences and Law appeared to be more vulnerable than students of Engineering and Architecture. A possible explanation for these results could be different difficulties and demands placed on students across various fields of study, which may have been reflected in their psychological distress.

Housing during the semester was also one of the important predictors of psychological problems during the COVID-19 pandemic. Living away from home was considered a risk factor for Internet addiction and depressive symptoms in both countries, and for anxiety symptoms in Slovak Republic. In these cases, students who lived away from home during the semester were more likely to have mental health problems compared to students who lived at home during the semester. This finding can be explained by distance from family support, infrequent contact, and less satisfaction with living in a dormitory (44, 45). In fact, parents and sufficient time spent with family members play an important role in students' wellbeing (44, 46). The results are consistent with those of Anand et al. (47), who found higher Internet addiction among students staying in rented accommodations. According to Romero-Rodríguez et al. (71), living outside the parents' house indicated higher rates of problematic Internet use, with parents at home showing a positive effect on reducing addictive online behaviors. Thus, the presence of parents at home may be an influential factor in terms of Internet addiction.

Regarding the distance between home and college, most of the analyzed cases were not significant. Slovak students who traveled between 50.1 and 100 km from home to college were less likely to be depressed compared to students who traveled more than 100 km. Also, Slovak students who traveled 20 km or less from home to college were less likely to be stressed than students traveling more than 100 km. Overall, the stated hypotheses H2a – H2e could be partially supported.

### Implications for Mental Health Policy

In the light of the presented findings, the need for increased mental health monitoring of higher education students can be emphasized. In this case, it appears crucial to identify effective interventions aimed at improving the mental health of Czech and Slovak youth during a difficult period in their lives, such as the COVID-19 pandemic. Professionals and policy-makers in both countries should therefore develop and implement strategies to effectively prevent mental health problems, including Internet addiction, anxiety, depression, as well as perceived stress. Overall, campaigns and programs should focus on full-time students and students living away from home during the semester. Problematic Internet use needs particular attention, as Internet addiction has been found to be associated with other problems that can further impair the mental state of young people. Therefore, efforts to help students addicted to the Internet are more than desirable. In this respect, the results showed that attention should be focused on full-time students, students living away from home during the semester, and students in Informatics, Mathematics, and ICT. With a reduction of Internet addiction, improvements in mental health in terms of anxiety symptoms, depressive symptoms, and stress can also be expected. Internet use should also be monitored during distance learning, when parents play an important role.

To improve the situation not only during the pandemic, a college-wide approach to students' mental health is needed in both countries, with teachers, assistants, professionals, and parents, but also policy-makers play an important role. It is crucial that Czech and Slovak policy-makers realize the importance of investing sufficient resources in special education and support for mental health care. In this sense, they should focus on strengthening the provision of early interventions and the development of open access mental health services for young people. Colleges and universities themselves should have the right conditions in place, including accessible counseling and equipping teachers with standardized mental health interviews to identify who needs help. Several colleges already have their own support centers that do activities to prevent and help students who need it. However, there are still colleges that do not provide such assistance to students or, on the other hand, do not have the capacity to help everyone. If a college does not have its own capacity, it is still possible to refer students to other organizations that provide this service, even virtually. Internet interventions in particular seem to have great potential in addressing this problem among today's college students. In this sense, a threat can turn into a help. The key is for colleges to communicate these opportunities. In the future, it is essential to collaborate more and help to improve the mental wellbeing of students studying in Czech Republic and Slovak Republic. Last but not least, the connections between colleges and families need to be improved. All of these efforts can lead to more intensive help, reduced stigma, and improved mental health literacy. In this respect, the COVID-19 pandemic can be seen as an opportunity to address a hitherto overlooked problem.

### Strengths and Limitations

The strengths of the study include in particular the large research sample in both countries (given their size) and the coverage of almost all universities and colleges, which increases the credibility of the results. This study also offers an in-depth insight into a serious social problem not only during the pandemic in the two countries that were directly compared. At this point, it should be emphasized that during the pandemic, most Slovak and Czech colleges opted for distance learning, which may increase the likelihood of internet addiction. Therefore, there is a need for research in this area to contribute to the knowledge and understanding of this phenomenon not only in the individual phases of the pandemic but also after the pandemic. Despite the importance of the issue, there is a lack of Slovak and Czech studies focusing on Internet addiction and its relationship with other mental health problems. Thus, this study contributes to addressing the limitations in the current literature by providing valuable findings and a better understanding of the issue. This can be seen as a strength from both a theoretical and practical point of view.

The study did not avoid certain limitations. The first limitation was that the approach to dealing with the pandemic in a higher education environment was not identical in the examined countries. Moreover, it was not the same even within individual colleges in one country. This may have created different conditions for students. In order to eliminate the risk of bias in the results due to different pandemic measures in the countries, the analyses were performed separately for Czech Republic and Slovak Republic. Sorting the analyses by individual colleges would not be practical, but although this step has not been taken, no significant bias in the data is expected. The second potential limitation was a certain disproportionality of the sample, as well as a lower number of observations in some categories. In this context, it should be noted that obtaining an ideal sample during a pandemic is considerably challenging. This is also due to the online survey, which has some limitations in capturing respondents. Given the situation, efforts have been made to obtain as representative a sample as possible. Regarding the lower number of observations, more caution is needed in interpreting the results, especially those related to the study field. Future research ambitions should focus on comparing the pandemic and post-pandemic periods in terms of the mental health problems of higher education students. Future research should also cover more countries.

## Conclusions

The Internet is an important service and is present in the lives of students for many purposes, such as gaining knowledge for study and having fun. On the other hand, it can be seen as a threat to their mental health, especially during distance learning. This study highlighted the importance of research on the mental health of higher education students during the COVID-19 pandemic in countries such as Czech Republic and Slovak Republic. This research revealed a high prevalence of mental health problems; confirmed the significant associations of Internet addiction with anxiety symptoms, depressive symptoms, and stress; as well as identified study-related characteristics associated with mental health problems in Czech and Slovak students. Therefore, the study provided a deeper insight into poor mental health as a growing concern among young people during the COVID-19 pandemic. Overall, the findings indicated that Internet addiction, anxiety, depression, and stress are issues that need to be addressed among higher education students from Czech Republic and Slovak Republic. There is a need to monitor their psychological problems, while full-time students and students living away from home require special attention. The valuable platform of results revealed by this research can help policy-makers and professionals in their efforts to improve the mental health of young people.

## Data Availability Statement

The raw data supporting the conclusions of this article will be made available by the authors, without undue reservation.

## Ethics Statement

The research was approved by the Ethics Committee of the General University Hospital in Prague as individual research (Ref. 915/20 S–IV). The study was conducted according to the guidelines of the Declaration of Helsinki. The participants provided their informed consent to participate in this study.

## Author Contributions

BG: conceptualization, writing—original draft preparation, writing—review and editing, visualization, supervision, project administration, and funding acquisition. SK: conceptualization, investigation, writing—original draft preparation, writing—review and editing, supervision, project administration, and funding acquisition. VI: conceptualization, methodology, investigation, resources, writing—original draft preparation, writing—review and editing, visualization, and supervision. MR: conceptualization, methodology, software, data curation, formal analysis, investigation, visualization, writing—original draft preparation, and writing—review and editing. TM: conceptualization, resources, writing—original draft preparation, writing—review and editing, visualization, supervision, and project administration. All authors contributed to the manuscript revision, read, and approved the submitted version.

## Funding

This research was funded by the Scientific Grant Agency of the Ministry of Education, Science, Research, and Sport of Slovak Republic and the Slovak Academy Sciences as part of the research project VEGA 1/0797/20: Quantification of Environmental Burden Impacts of the Slovak Regions on Health, Social and Economic System of the Slovak Republic. This research was supported by the Slovak Research and Development Agency under the contract No. APVV-17-0360: Multidimensional analysis of significant determinants of public procurement efficiency with emphasis on the application of Health Technology Assessment in the procurement preparation phase.

## Conflict of Interest

The authors declare that the research was conducted in the absence of any commercial or financial relationships that could be construed as a potential conflict of interest.

## Publisher's Note

All claims expressed in this article are solely those of the authors and do not necessarily represent those of their affiliated organizations, or those of the publisher, the editors and the reviewers. Any product that may be evaluated in this article, or claim that may be made by its manufacturer, is not guaranteed or endorsed by the publisher.
