# Supplementary material for: Internet Addiction, Symptoms of Anxiety, Depressive Symptoms, Stress Among Higher Education Students During the COVID-19 Pandemic
Source: Front Public Health. 2022 Jun 14;10:893845. doi: 10.3389/fpubh.2022.893845 (PMC9237380; doi:10.3389/fpubh.2022.893845)
Supplement: Supplementary file 1 [file Data_Ssheet_1.docx]

Supplementary Material

**Supplementary Table 1.** Internet Addiction Test (IAT) – Czech and Slovak version

| ID | CZ | SK |
| --- | --- | --- |
| 1 | Jak často zjistíte, že jste zůstali online déle, než jste chtěli? | Ako často zistíte, že zostávate online dlhšie, ako ste plánovali? |
| 2 | Jak často zanedbáváte domácí práce, abyste mohli být více času online? | Ako často zanedbávate domáce práce, aby ste trávili viac času online? |
| 3 | Jak často upřednostňujete vzrušení na internetu před intimitou se svým partnerem? | Ako často uprednostňujete vzrušenie na internete pred intimitou so svojím partnerom? |
| 4 | Jak často navazujete nové vztahy s ostatními online uživateli? | Ako často vytvárate nové vzťahy s ostatnými online používateľmi? |
| 5 | Jak často Vám lidé ve vašem životě vyčítají délku času, který trávíte online? | Ako často sa vám ostatní vo vašom živote sťažujú na množstvo času, ktorý trávite online? |
| 6 | Jak často zanedbáváte školní povinnosti kvůli času, který trávíte online? | Ako často zanedbávate školské povinnosti kvôli času, ktorý trávite online? |
| 7 | Jak často kontrolujete svůj mail předtím, než se pouštíte do nového úkolu? | Ako často kontrolujete svoj e-mail pred tým, než sa pustíte do nejakej novej úlohy? |
| 8 | Jak často internet negativně ovlivňuje váš pracovní výkon, nebo Vaši produktivitu? | Ako často internet negatívne ovplyvňuje váš pracovný výkon, alebo produktivitu? |
| 9 | Jak často jste uzavření nebo děláte tajnosti, když se Vás někdo zeptá, co děláte na internetu? | Ako často sa uzatvárate do seba, alebo správate tajnostkársky, keď sa vás niekto spýta, čo robíte online? |
| 10 | Jak často nahrazujete svoje nepříjemné myšlenky o svém životě, uspokojujícími myšlenkami z internetu? | Ako často blokujete rušivé myšlienky o svojom živote upokojujúcimi myšlienkami z internetu? |
| 11 | Jak často myslíte na to, kdy už budete online? | Ako často sa zamýšľate nad tým, kedy budete znova online? |
| 12 | Jak často se obáváte, že život bez internetu by byl nudný, nenaplněný a neutěšený? | Ako často sa obávate, že život bez internetu by bol nudný, nenaplnený a neutešený? |
| 13 | Jak často Vás naštve nebo se nazlobíte, když vás někdo vyrušuje, když jste online? | Ako často vás naštve alebo ste nahnevaný, keď vás niekto vyrušuje počas toho, ako ste online? |
| 14 | Jak často se nevyspíte dobře, když jste byli dlouho do noci online? | Ako často sa nevyspíte dobre, kvôli tomu, že ste online dlho do noci? |
| 15 | Jak často se cítíte znepokojení, když jste offline, anebo myslíte na to, kdy už budete online? | Ako často sa cítite znepokojený, keď ste offline, alebo myslíte na to že budete online? |
| 16 | Jak často říkáte "už jen chvilku", když jste online? | Ako často si hovoríte "už len chvíľu", keď ste online? |
| 17 | Jak často se snažíte zkátit si čas, když jste online a nedaří se Vám to? | Ako často sa snažíte skrátiť čas strávený online a nepodarí sa vám to? |
| 18 | Jak často se snažíte zakrývat to, že jste online? | Ako často sa snažíte skrývať čas strávený online? |
| 19 | Jak často si vyberete možnost být online před tím, když můžete jít ven? | Ako často si vyberiete byť online pred možnosťou ísť niekam von? |
| 20 | Jak často se cítíte depresivně, jste náladový nebo nervózní, když jste offline a tento stav zmizí ve chvíli, když jste zase online. | Ako často sa cítite depresívne, ste náladový alebo nervózny, keď ste offline a tento stav zmizne vo chvíli, keď ste späť online. |

Note: Wording of the item – CZ: Vyjádřete svůj postoj k následujícím položkám; SK: Vyjadrite svoj postoj k nasledujúcim položkám.

**Supplementary Table 2.** Patient Health Questionnaire (PHQ-9) – Czech and Slovak version

| ID | CZ | SK |
| --- | --- | --- |
| 1 | Malý zájem anebo potěšení dělat věci | Malý záujem alebo potešenie robiť veci |
| 2 | Depresívní pocit anebo pocit beznaděje | Depresívny pocit alebo pocit beznádeje. |
| 3 | Problémy s usínáním anebo s délkou spánku | Problémy so zaspávaním alebo s dĺžkou spánku |
| 4 | Pocit únavy anebo nedostatku energie | Pocit únavy alebo nedostatku energie |
| 5 | Nechuť do jídla nebo přejídání se | Zlá chuť do jedla alebo prejedanie sa |
| 6 | Pocit selhání | Pocit zlyhania |
| 7 | Problémy se soustředěním, jako např. při čtení, sledování televize, poslouchání jiné osoby při vzájemném rozhovoru a pod. | Problémy s koncentráciou, ako napr. pri čítaní, pozeraní televízie, počúvaní inej osoby pri vzájomnom rozhovore a pod. |
| 8 | Pohybujete se anebo hovoříte tak pomalu, že si toho ostatní mohli všimnout, anebo naopak - rychle? | Pohybujete sa alebo hovoríte tak pomaly, že si to ostatní mohli všimnúť, alebo naopak - rýchlo? |
| 9 | Měli jste myšlenky, že by bylo lepší umřít, anebo si nějak ublížit | Mali ste myšlienky, že by bolo lepšie umrieť, alebo si nejako ublížiť |

Note: Wording of the item – CZ: Trápili Vás během posledních 2 týdnů nějaké z následujících těžkostí?; SK: Trápili Vás počas posledných 2 týždňov nejaké z nasledujúcich ťažkostí?

**Supplementary Table 3.** Generalized Anxiety Disorder (GAD-7) – Czech and Slovak version

| ID | CZ | SK |
| --- | --- | --- |
| 1 | Pocit nervové únavy anebo na hraně Vašich možností | Pocit nervovej únavy alebo na hrane Vašich možností |
| 2 | Neschopnost zastavit anebo kontrolovat obavy | Neschopnosť zastaviť alebo kontrolovať obavy |
| 3 | Přílišné obavy | Prílišné obavy |
| 4 | Problémy s odpočinkem | Problémy s oddychom |
| 5 | Nepokojnost do té míry, kdy je problematické klidně sedět | Nepokojnosť do miery, kedy je problematické pokojne sedieť |
| 6 | Snadno jste se nazlobili | Ľahko ste sa nahnevali |
| 7 | Pocit strachu, že by se mohlo stát něco strašného | Pocit strachu, ako by sa mohlo stať niečo strašné |

Note: Wording of the item – CZ: Trápili Vás během posledních 2 týdnů nějaké z následujících těžkostí?; SK: Trápili Vás počas posledných 2 týždňov nejaké z nasledujúcich ťažkostí?

**Supplementary Table 4.** Perceived Stress Scale (PSS) – Czech and Slovak version

| ID | CZ | SK |
| --- | --- | --- |
| 1 | Jak často jste se cítili rozrušeně, protože se stalo něco nečekaného? | Ako často ste sa cítili rozrušený, pretože sa stalo niečo nečakané? |
| 2 | Jak často jste měli pocit, že nejste schopni kontrolovat důležité věci ve svém životě? | Ako často ste mali pocit, že nie ste schopný kontrolovať dôležité veci vo Vašom živote? |
| 3 | Jak často jste byli nervózní a stresovaní? | Ako často ste boli nervózny a stresovaný? |
| 4 | Jak často jste byli přesvědčení o své schopnosti zvládat svoje osobní problémy? | Ako často ste boli presvedčený o svojej schopnosti zvládať svoje osobné problémy? |
| 5 | Jak často jste měli pocit, že věci se vyvíjejí podle Vašich představ? | Ako často ste mali pocit, že veci sa vyvíjajú podľa Vašich predstáv? |
| 6 | Jak často jste zjistili, že nemůžete zvládat všechno, co jste měli udělat? | Ako často ste zistili, že nie ste schopný zvládnuť všetko, čo ste mali urobiť? |
| 7 | Jak často jste kontrolovali iritující podněty? | Ako často ste kontrolovali iritujúce podnety? |
| 8 | Jak často jste měli pocit, že jste "na koni" a daří se Vám? | Ako často ste mali pocit, že ste "na koni" a darí sa Vám? |
| 9 | Jak často jste byli nazlobeni kvůli věcem, které jste nemohli ovlivnit? | Ako často ste boli nahnevaný kvôli veciam, ktoré ste nemohli ovplyvniť? |
| 10 | Jak často jste cítili, že že máte tolik těžkostí, že je nepřekonáte? | Ako často ste cítili, že ťažkosti sa tak nakopili, že ich neprekonáte? |

Note: Wording of the item – CZ: Jaký je Váš postoj k následujícím výrokům?; SK: Aký je Vás postoj k nasledujúcim výrokom?
